# Inferior Vena Cava Edge Tracking Echocardiography: A Promising Tool with Applications in Multiple Clinical Settings

**DOI:** 10.3390/diagnostics12020427

**Published:** 2022-02-07

**Authors:** Stefano Albani, Luca Mesin, Silvestro Roatta, Antonio De Luca, Alberto Giannoni, Davide Stolfo, Lorenza Biava, Caterina Bonino, Laura Contu, Elisa Pelloni, Emilio Attena, Vincenzo Russo, Francesco Antonini-Canterin, Nicola Riccardo Pugliese, Guglielmo Gallone, Gaetano Maria De Ferrari, Gianfranco Sinagra, Paolo Scacciatella

**Affiliations:** 1 Division of Cardiology, Umberto Parini Regional Hospital, 11100 Aosta, Italy; lbiava@ausl.vda.it (L.B.); cbonino@ausl.vda.it (C.B.); lcontu@ausl.vda.it (L.C.); epelloni@ausl.vda.it (E.P.); pscacciatella@ausl.vda.it (P.S.); 2 Cardio-Thoraco-Vascular Department, Division of Cardiology and Postgraduate School in Cardiovascular Sciences, University of Trieste, 34127 Trieste, Italy; deluca.antonio.md@gmail.com (A.D.L.); davide.stolfo@gmail.com (D.S.); gianfranco.sinagra@asugi.sanita.fvg.it (G.S.); 3 Mathematical Biology & Physiology, Department of Electronics and Telecommunications, Politecnico di Torino, 10129 Torino, Italy; luca.mesin@polito.it; 4 Integrative Physiology Lab, Department of Neuroscience, University of Turin, 10125 Turin, Italy; silvestro.roatta@unito.it; 5 Scuola Superiore Sant’Anna, 56127 Pisa, Italy; a.giannoni@santannapisa.it; 6 Fondazione Toscana G. Monasterio, 56124 Pisa, Italy; 7 Department of Translational Medical Sciences, University of Campania Luigi Vanvitelli-Monaldi Hospital—A.O.R.N. Dei Colli, 80131 Naples, Italy; emilioattena@hotmail.it (E.A.); v.p.russo@libero.it (V.R.); 8 Highly Specialized in Rehabilitation Hospital—ORAS S.p.A. of Motta di Livenza, 31045 Treviso, Italy; Francesco.AntoniniCanterin@ospedalemotta.it; 9 Department of Clinical and Experimental Medicine, University of Pisa, 56126 Pisa, Italy; n.r.pugliese88@gmail.com; 10 Division of Cardiology, Città della Salute e della Scienza, University of Turin, 10124 Turin, Italy; guglielmo.gallone@gmail.com (G.G.); gaetanomaria.deferrari@unito.it (G.M.D.F.)

**Keywords:** inferior vena cava, right atrial pressure, caval index, heart failure, pulmonary hypertension, edge tracking

## Abstract

Ultrasound (US)-based measurements of the inferior vena cava (IVC) diameter are widely used to estimate right atrial pressure (RAP) in a variety of clinical settings. However, the correlation with invasively measured RAP along with the reproducibility of US-based IVC measurements is modest at best. In the present manuscript, we discuss the limitations of the current technique to estimate RAP through IVC US assessment and present a new promising tool developed by our research group, the automated IVC edge-to-edge tracking system, which has the potential to improve RAP assessment by transforming the current categorical classification (low, normal, high RAP) in a continuous and precise RAP estimation technique. Finally, we critically evaluate all the clinical settings in which this new tool could improve current practice.

## 1. Introduction

An elevated right atrial pressure (RAP) predicts a poor outcome in patients with heart failure (HF) [1], and it is an important target to optimize diuretic and venodilator treatments in this setting. Therefore, precise RAP estimation has important clinical and therapeutical implications. The inspiratory collapse of the inferior vena cava (IVC) and the measurement of its diameters during the respiratory cycle are widely used in clinical practice for RAP estimation [2], but the correlation between RAP assessed invasively and by echocardiography, and the reproducibility of IVC assessment by ultrasound (US), are no more than modest [3,4,5,6,7]. Specifically, the current technique provides fair accuracy when estimating low or high RA pressures, but it remains inaccurate to estimate intermediate values that encompass most patients across a range of clinical conditions [4] (Table 1). Moreover, the dichotomic output of IVC-based estimates (i.e., low or high RAP) fails to represent the continuous range of RAP values that may entail important therapeutic and prognostic implications. In this manuscript, we describe solutions that might improve the reliability and reproducibility of IVC assessment by echocardiography to correctly assess RAP values.

## 2. Physiological Dynamic Changes in IVC Size

Due to its high compliance, IVC promptly changes its size in response to changes in transmural pressure. In particular, changes in IVC transmural pressure are regularly produced by respiratory activity: during spontaneous breathing, IVC size decreases in the inspiratory phase due to decreased intrathoracic pressure, which favors blood flow to the thorax and increased abdominal pressure, as compared to expiration [8]. Conversely, increased IVC size during inspiration is observed in ventilated patients due to the opposite-sign pressure changes taking place in the thorax [8]. Importantly, any maneuver or pathological condition that alters blood pressure and volume in the abdominal compartment will not only affect the IVC size but also the magnitude of size changes (e.g., respiratory oscillations), since vessel compliance decreases with size. On this basis, the alteration of RAP and volemic status are often evidenced by quantification of phasic changes in vessel size by means of pulsatility indices (e.g., caval index, collapsibility index, distensibility index, used as synonyms) generally calculated as the ratio $\frac{\mathrm{Dmax}-\mathrm{Dmin}}{\mathrm{Dmax}}$, D_max_, and D_min_ being the maximum and minimum diameter observed in a respiratory cycle, respectively. However, the clinical reliability of these indices is still debated [9,10,11].

In this respect, two recent findings will be emphasized. First of all, here, we intendedly refer to IVC size rather than diameter, since the latter is inadequate to describe the IVC, often exhibiting a non-circular cross-section and anisotropic deformation during both spontaneous breathing and fluid challenges [12,13,14,15]. Although the standard M-mode ultrasound approach necessarily generates a mono-dimensional monitoring of IVC size, automated methodologies now offer the possibility to account for changes in the full cross-sectional area while still expressing the results in terms of “equivalent diameter” and adopting the same pulsatility indices [14,15].

**Figure 1:**
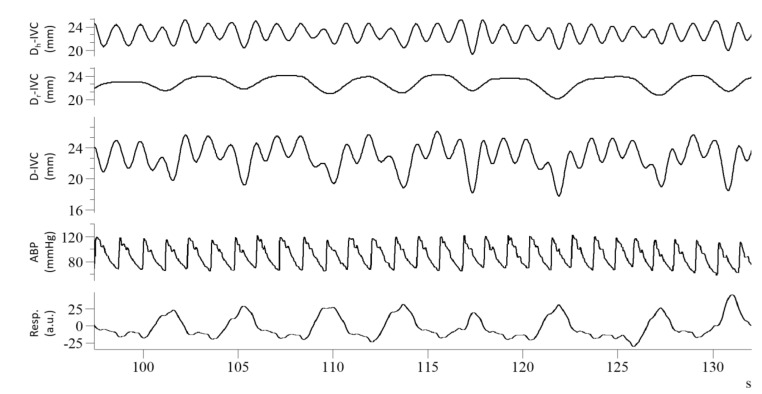
Recordings from a healthy subject: from top to bottom, cardiac component of IVC pulsatility (Dc-IVC), respiratory component (Dr-IVC), unfiltered IVC pulsatility (D-IVC), arterial blood pressure (ABP), respiratory movements (Resp), long axis IVC imaging was processed according to Mesin et al., 2019 [24].

A second issue concerns the nature of physiological oscillations in IVC size. It was recently pointed out that a pulsatility of cardiac origin superimposes on the previously mentioned respiratory oscillations [14,16,17,18,19]. In fact, the pulsatility that characterizes RAP is transmitted backwards to the venous compartment and can be observed non-invasively in major veins (e.g., the superior and inferior vena cava, the internal jugular vein, the hepatic vein) with different imaging modalities, including US [20], Doppler US [21], and, more recently, photoplethysmography [22] and magnetic resonance [23]. The cardiac contribution to the pulsatility of IVC size (i.e., the variation in IVC diameter during a cardiac cycle) has been generally ignored or neglected due to the difficulty of discriminating cardiac and respiratory components with traditional techniques. However, in our preliminary investigations in healthy subjects and patients, we observed that filtering out the cardiac component reduces the caval index (CI) by 40–50% [15,24]. Exclusively considering the cardiac pulsatility, by means of the *cardiac caval index*, has been suggested to address a major limitation of the CI: its high temporal variability, which is derived from the intrinsic variability of spontaneous respiration [17,19]. In a recent study, we pointed out that even the isolated cardiac pulsatility of IVC maintains a residual modulation of respiratory nature, as can be noticed in the first tracing of Figure 1, and that further filtering-out this modulation significantly improves intra-subject reliability during long-duration IVC monitoring [25].

In addition, the cardiac pulsatile component may carry different and complementary information to the respiratory one [16]. It is interesting to observe that its magnitude is highly variable among different subjects and exhibits little intra- and inter-subject correlation with the magnitude of the respiratory component [19,23,25]. Indeed, IVC cardiac pulsatility was shown to improve patient classification with respect to volume status [15] and RAP [26]. For these reasons, assessment of this new index is promising and deserves further investigation to assess its usefulness in the clinical routine.

## 3. Critical Issues in RAP Assessment Using IVC

The literature and guidelines report a lack of standardization of the measurement of IVC site, ranging from 5 to 30 mm from the right atrial junction and, in most studies, there is no agreement on which site is the most reliable [3,27]. The identification of a reference point to guide the measurement is another critical aspect [28]. Indeed, a recent study showed that IVC has large variations in pulsatility along the longitudinal axis, suggesting that it is better to average across an entire portion of the vessel instead of focusing on a single section [24]. According to some authors, the junction between the IVC and right atrium is characterized by lower compliance compared to a more distal site of the IVC [3]. This could have clinical implications: an IVC measurement at the distal part could provide different information compared to a more proximal analysis. Moreover, M-Mode measurements may be inaccurate in this setting due to the difficulties in proper alignment perpendicularly to the vessel [3]. In addition, the use of the “sniff” maneuver to estimate the inspiratory collapse is questionable. Indeed, the inspiratory “sniff” is poorly objectifiable, resulting in a low reliability, depending on the patient features [29]. Other authors have highlighted how, during the act of breathing, the IVC moves in the cranio-caudal and latero-lateral directions [12]. Such displacements could be able to influence the reproducibility and reliability of the measurement [19,30]. Moreover, the type of breathing ranges from superficial to deep and requires efforts from diaphragmatic to thoracic [18], and this aspect could certainly play a role in RAP assessment through IVC diameters [29]. Finally, one group has even discouraged the use of the IVC with the purpose of estimating and stratifying RAP in various ranges of values due to the high inaccuracy reported in a sample of 200 patients undergoing right heart catheterization (RHC) [31]. Finally, even in the field of intensive care unit, in mechanically ventilated patients, the US assessment of the IVC has shown several limitations about its clinical usefulness [11]. In conclusion, current techniques on RAP assessment using IVC diameters have several limitations, and new tools are needed.

## 4. Standardization of RAP Measurement

**Figure 2:**
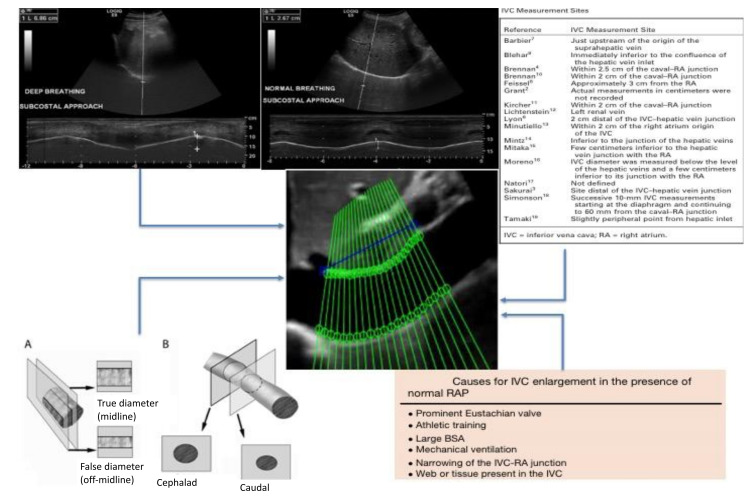
Critical issues on RAP estimation by using IVC diameters. **Top left**: different breathing manners studied with US M-mode of the diaphragm. **Top right**: different proposed sites of measurement of the IVC. **Bottom left**: effect of the vein movement on the IVC diameter measurement showing foreshortening of the vein due to the respiratory cycle (A: latero-lateral displacement, B: cranio-caudal displacement) [12]. **Bottom right**: causes of unreliability of the IVC in RAP estimation [2]. Center: the proposed new IVC edge-to-edge tracking technique (adapted from Blehar et al. [12] and Wallace et al. [27]).

An option to standardize the measurement of the IVC and to make the estimation of RAP more accurate and reproducible could be the use of a software able to automatically highlight the edges of the vessel, i.e., an “edge-tracking” technique [19]. Both the long and short axis view have been investigated by our research group [14,24]. While different methods have been presented in the literature (long axis [17], short axis [16,32,33], the current dissertation is limited to those techniques more efficient in terms of computational costs (that could thus be promptly implemented in clinical practice) and with stable data processing [14,32]. Several potential advantages may be present with our suggested methods. First, they compensate for the movements of the IVC by investigating the displacement directions of the vein during its movements. In fact, different collapsibility is shown by IVC in different directions [14,19,24] due to local variations of compliance, which are also influenced by external tissues. Second, an entire portion of the IVC is considered (either in the axial direction or in the cross-section, for the long and short axis views, respectively). Indeed, while possible noise or artifacts could invalidate the estimations in specific frames and directions, averaging information across a portion of the IVC could better reflect its behavior and stabilize the extracted information. This way, the diameter of each site could be measured to accurately detect even slight variations in the caliber of the vein (Figure 2).

**Figure 3:**
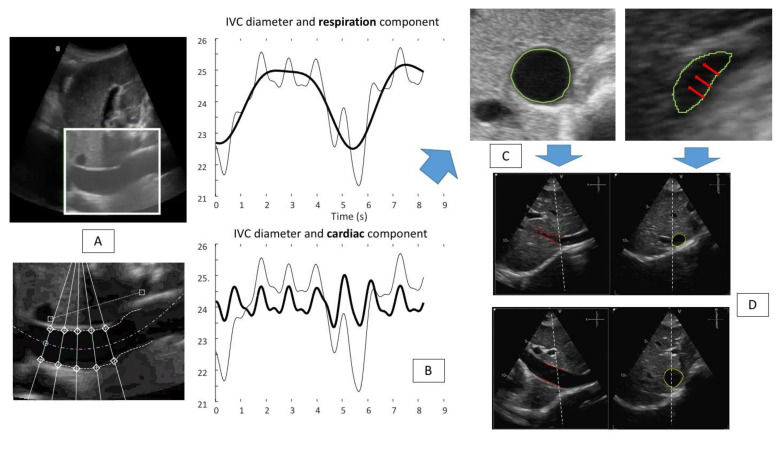
Panel (**A**): IVC tracking technique: the software is able to identify the vessel (IVC long axis view) (**top**) of interest and perform the edge tracking of five fixed points along both edges of the vessel (**bottom**). Panel (**B**): the results of the edge tracking techniques are shown: respiratory (**top**) and cardiac (**bottom**) components are represented. (Bold line graphic on top: respiratory component of the IVC diameter variation (mm). Bold line graphic at the bottom: cardiac component of the IVC diameter variation (mm). Non-bold line in both graphics: IVC diameter variation during two breaths). Panel (**C**): a US IVC scan in short axis view is shown; red arrows indicate the direction of the collapsibility of the vessel walls that is not directed in an antero-posterior way, rather the main direction is medio-lateral. Panel (**D**) shows a possible solution to perform a more reliable IVC study: the use of the three-dimensional x-plane echocardiography, to gain the possibility to evaluate in real-time both long and short axis views (adapted from Mesin et al. [8]).

This information was used to automatically provide an estimation of RAP during the resting breath to avoid “breathing bias” [29,30]. Specifically, the estimated IVC diameter was post-processed evaluating the physiological oscillatory components of the IVC induced both by the cardiac and the respiratory cycles (Figure 3, panel A and B) [29]. Currently, this method has been clinically tested in 49 patients undergoing RHC for clinical reasons [26,29].

Several models built on CI, the pulsatility indicators reflecting either respiratory or cardiac stimulation and the mean diameter of the IVC, together with anthropometric data were tested against invasively measured RAP. The best model showed high agreement to estimate RAP as a continuous variable (mean error 3.6 ± 2.6 mm Hg) [26]. Then, a binary tree model was developed to classify RAP according to different range of values [29]. This model estimated invasive RAP with high accuracy (R^2^ = 0.61) and proved superior to standard US IVC-based methods [29].

**Figure 4:**
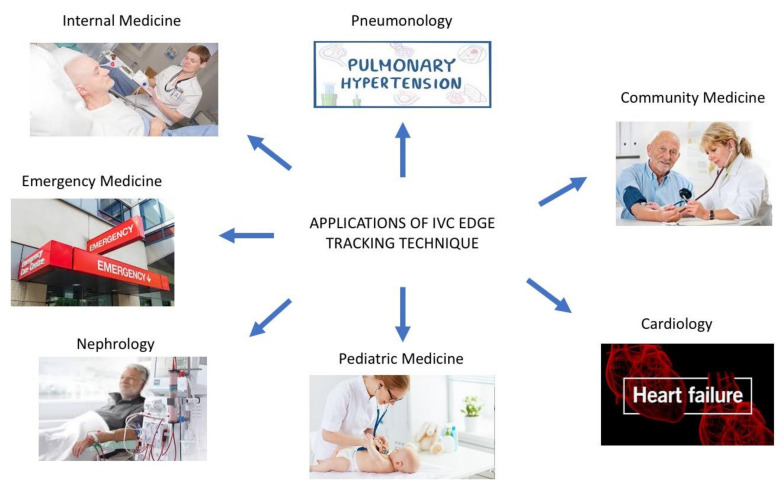
Our edge tracking technique could be applied in multiple clinical settings (*see the text for further explanations*).

The integration of our semiautomated method with the three-dimensional ultrasound probe, with the x-plane technique made available through three-dimensional US probes may further refine RAP estimation (Figure 3 panel D) [34]. Indeed, as shown in Figure 3 panel C, the direction of the collapsing walls of the IVC could happen in different planes rather than only the ante-posterior one. The relevance of this aspect was recently demonstrated by our group [14]. While standard 3D US IVC full volume-based RAP assessment was recently demonstrated to have high accuracy by Huguet et al. [35], our new semiautomated tool may provide a user-friendly tool to be used also by non-expert operators of cardiac imaging laboratories. Indeed, our tool may provide non-experienced operators, who have to assess the volume status of the patients, with a quick, easy to use, reliable diagnostic technique to be used across a variety of clinical settings (Figure 4), even in the contexts in which a multi-parameter assessment could be challenging and not functional.

## 5. RAP as a Marker of Congestion: Current Advanced Technique of Congestion Assessment and Prognostic Implications

In ambulatory patients with HF, the estimation of RAP by echocardiography is a powerful prognostic index of early hospitalization or death [1]. A distended IVC is highly prevalent in patients with few signs or symptoms of HF and identifies those at greater risk [36].

The assessment of congestion is a critical aspect in HF management. Indeed, patients admitted for HF worsening with residual congestion at discharge presented higher risk of mortality and readmission [37,38,39]. The clinical evaluation alone has low accuracy to detect congestion [26], and several clinical, laboratoristic, and imaging parameters are suggested as decongestion targets with the aim to improve a patient’s outcomes [40].

**Figure 5:**
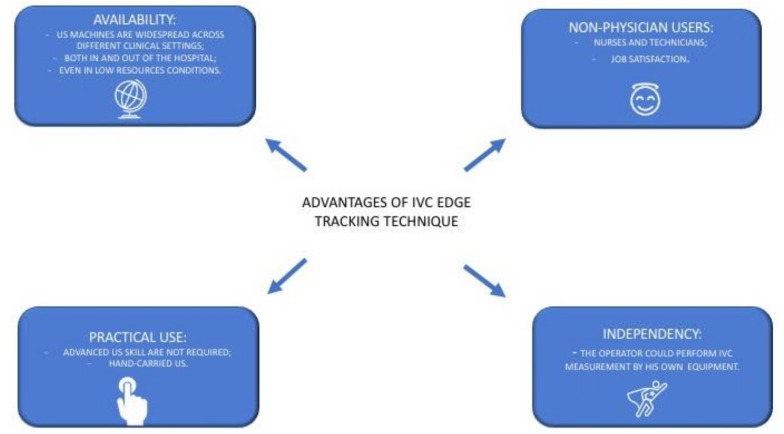
Main advantages that could be provided by an extensive use of US IVC edge tracking echocardiography (*see text for further explanations*).

The IVC is one of the most studied echocardiographic parameters in this field [41], and its expiratory diameter is strictly related with NTproBNP, which has the prognostic ability to predict all-cause mortality at 1 year [42]. Moreover, a recent study has shown that the end expiratory IVC diameter has relevant predictive ability independent from other well-known prognostic markers in HF, including NTproBNP itself [36]. These findings suggest that the IVC expiratory diameter and NTproBNP could play a complementary role in prognostic HF stratification both in the preserved and reduced ejection fraction settings [42]. There are ongoing trials investigating if a US IVC guided decongestion strategy could offer prognostic advantages over current standard of care [43]. We speculate that our algorithm may improve IVC-guided decongestion strategies by providing a more accurate method to detect subtle changes in RAP intra-patient variations thanks to its ability to detect even small collapsibility variations of the vessel during resting respiration. Several advantages of this new “echocardiographic marker” of congestion may be hypothesized to guide clinical management across a range of medical settings (Figure 5).

(1) Availability: The IVC measurement only needs a US machine with sector/convex probes that are available in many clinical settings, both in and out of the hospital, even in low resources settings [44]. Indeed, IVC edge tracking echocardiography is a low-cost solution that only needs the acquisition of a new software to be ready for clinical use.
(2) Practicality: The IVC assessment could be performed easily by an operator with limited US experience and with a hand-held US machine [45,46]. No adjunctive or specific training is required.
(3) Adoption by non-physicians users: Trained nurses may successfully use US for IVC assessment [47]. Thus, IVC edge tracking echocardiography may be easily managed by nurses, giving them more autonomy and personal skills. This aspect could have a foreseeable impact on resource optimization and it could contribute to fight against nurses’ widespread job dissatisfaction [48].
(4) Autonomy: The physician that works in an outpatient chronic HF clinic approaches the patients with different management models across different countries [49]. Indeed, the assessment of cardiac biomarkers of congestions may not always be readily available to guide HF management [50]. Conversely, the edge tracking technique for IVC diameter assessment could be quickly performed by the physician himself during the ambulatory evaluation or by trained nurses, as mentioned above, independently from other services (such as the laboratory department for the biomarker dosage).

## 6. RAP in Advanced Heart Failure and Pulmonary Hypertension

In the setting of advanced HF, RAP may reflect right ventricular function. Thus, a precise RAP estimation may be of importance to evaluate right ventricular function, especially in candidates for a left ventricular assist device (L-VAD). The Right Ventricle Stroke Work Index (RVSWI) is a hemodynamic index that has been shown to be a reliable parameter in the selection of these patients [51]. However, this index is only available after RHC. Some authors tried to convert the information from the RVSWI with an echocardiographic index, the Right Ventricle Contraction Pressure Index (RVCPI). However, a positive but not optimal correlation emerged from their study, which is mainly due to poor reliability in predicting RAP [52]. The same group proposed a new algorithm to predict RAP with exceptional accuracy (r^2^: 0.70) [53], this index (estimated RAP, eRAP) consists of the mean of the available values of RAP derived by the IVC assessment [3], the hepatic venous flow pattern (hepatic venous systolic-to-diastolic wave ratio), and the hepatic venous systolic filling fraction defined as $\frac{\mathrm{systolic}\;\mathrm{VTI}}{\mathrm{systolic}\;\mathrm{VTI}\;+\;\mathrm{diastolic}\;\mathrm{VTI}}$ (ratio of systolic and diastolic hepatic venous velocity–time integrals—VTIs) [54]. This study, as others [54], in fact proposed the use of hepatic venous Doppler parameters as a cornerstone to correctly estimate RAP. Indeed, in both studies, interobserver variability was performed by expert operators: in the former, the authors did not specify in how many patients the index was calculated by the second operator, whereas in the latter study, the reliability was calculated in only 10 patients [53,54]. Other studies showed only moderate reproducibility [55] of hepatic vein Doppler to predict RAP, and recently, some authors failed to reproduce the proposed eRAP index in a cohort of patients who underwent RHC. They conclude that the multiparametric eRAP index does not provide an advantage over guidelines suggested for RAP assessment, despite being more complex and time consuming [56]. In the field of advanced heart failure, it is of paramount importance to stress that our tool is able to predict right heart chambers congestion only, on the other hands, there are several echocardiographic techniques to predict left ventricular end diastolic pressure, which is another important parameter to know how to correctly manage such advanced patients [1].

Finally, RAP is an important treatment target in pulmonary arterial hypertension, where elevated values are a proxy of a failing right ventricle heralding adverse prognosis [57,58]. A lot of prognostic markers have been studied in such patients; however, due to a prevalence of pneumologists as clinical managers of such a disease, echocardiographic parameters have been evaluated only in a few studies compared to other clinical variables [59,60,61]. Our new system could broaden the adoption of serial RAP assessment as a prognostic marker and treatment target among patients with advanced HF and pulmonary arterial hypertension [46], enhancing current RAP grading with its continuous nature.

## 7. Usefulness of IVC Edge Tracking Technique at the Emergency Department

HF with reduced ejection fraction (HFrEF) is one of the most studied conditions in cardiology, and many pharmacological and non-pharmacological treatments proved their efficacy in this condition [62]. Nevertheless, many patients are admitted to cardiology departments for the relapse of HF each year [63]. On the other hand, heart failure with preserved ejection fraction (HFpEF), although as frequent as HFrEF [64], remains an unmet need in the cardiology field in terms of treatment efficacy. Indeed, the recommended treatment strategy of HFpEF involves blood pressure control, low salt diet, and the reduction of the impact of cardiovascular risk factors, such as smoking, diabetes, dyslipidemia, and obesity as the recommended treatment [65]. Diuretic therapy is the cornerstone of medical management of such a condition; however, no studies have demonstrated their efficacy on long-term outcomes [66]. Due to the lack of an efficient treatment, patients affected by this condition are treated for their relapsing events at the Emergency Department and then in Internal Medicine wards, with an important economic impact caused by their long hospital stay and their frequent multiple admissions for HF relapse [67,68]. Therefore, the clinical goals to treat this condition are to reduce the in-hospital stay and to avoid clinical relapse of HF. The former condition was extensively studied using both biomarker-guided therapy and US assessment, which demonstrated inconclusive results. Indeed, the systematic use of biomarkers or the IVC assessment are not routinely recommended to treat these patients [69,70,71,72].

In the latter situation, several studies were performed to follow these patients with periodical nurse assessment, to tailor diuretic therapy using weight assessment or NTproBNP strategy, and even in this context, no recommendations were available due to the weakness of results [73,74]. We speculate that our edge tracking technique could be able to precisely assess the volume status of the patients affected by this condition and lead the clinicians accurately in both settings. Specifically at the Emergency Department, our tool could help the emergency physicians to optimize diuretic therapy providing objective and reliable data to manage patients affected by heart failure in terms of dosage of diuretic administration and diuretic response to treatment. All of this information could be used in the decision-making process of the patient management ward admission vs. early discharge vs. treatment in the emergency department observation unit.

On the other hand, in the out-patient ambulatory office, the general practitioner (helped by community nurses) could follow patients affected by chronic heart failure himself with the theoretical possibility of avoiding some access to the emergency department with a robust and reliable parameter to rely on.

## 8. IVC in Children with Nephrotic Syndrome

Generalized edema is one of the main causes of hospitalization of children with nephrotic syndrome and, if not appropriately treated, may lead to death [75]. Indeed, there is evidence that suggests that some of these patients developed congestive edema, and in this case, diuretic therapy should be started [76]. The assessment of the IVC as a marker of fluid overload has been investigated in many studies, which report conflicting results. Some authors found positive correlation of the IVC Caval Index (IVCCI), which is defined as $\frac{\mathrm{IVC}\;\mathrm{expiratory}\;\mathrm{Diameter}\;-\;\mathrm{IVC}\;\mathrm{inspiratory}\;\mathrm{Diameter}}{\mathrm{IVC}\;\mathrm{expiratory}\;\mathrm{Diameter}}$, with the intravascular volume [77,78]. However, these studies included only small cohorts of patients, and the gold standard used was not robust, conferring important weakness to the authors’ conclusions. On the other hand, other authors found that IVCCI was not adequate to help in the identification of clinical subgroups of patients affected by nephrotic syndrome [79]. Buyukavci et al. found that IVCCI was not significantly different between hypovolemic and non-hypovolemic patients classified using fractional sodium excretion rate [80]. Finally, another group documented that bioimpedance measures may be superior to IVCCI in determining volume load in children with nephrotic syndrome [81].

The sniff maneuver could not be performed to evaluate the collapsibility of the IVC in children due to the lack of collaboration of the youngest [82] and the probable low reliability rate of the oldest that could be evaluated as adults [27]. We think that our edge tracking technique could positively improve fluid assessment in children due to the lack of use of the sniff maneuver. Moreover, US is a radiation-free technique that is wide available in the majority of pediatric departments, without contraindications in the diagnostic evaluation in children [83].

## 9. IVC Assessment in Patients Undergoing Dialysis

IVC assessment to identify patients with fluid overload before the dialysis session is a validated technique to assess fluid overload [45,84,85]. However, fluid removal guided by IVC US assessment to prevent post-dialysis hypotensive episodes reported conflicting results, which was possibly due to the low accuracy of current IVC US assessment to predict volemic status [45,86,87]. The proposed edge tracking technique may improve volemic assessment in patients undergoing dialysis predicting the correct amount of volume depletion prior to dialysis and allowing to monitor and adjust the dialysis parameters during the treatment. Moreover, even in a nephrological setting, the feasibility and reliability to perform the IVC US assessment by non-physicians was demonstrated with acceptable interrater agreement [87].

## 10. Other Techniques for RAP Assessment

The rate of the US evaluation of the IVC is 80% in the majority of available studies [4,29,31]. In the residual 20% of patients, other indexes should be considered. In a study with 200 patients, distension of the jugular vein at rest relative to the maximum diameter during a Valsalva maneuver (JVD ratio), assessed by vascular US, identified patients with heart failure who had higher plasma NTproBNP levels, right ventricular impairment, and raised pulmonary artery pressure [88]. In another study of 243 patients, near-infrared spectroscopy was used to estimate RAP, identifying ambulatory patients with chronic heart failure who had more severe congestion and a worse outcome [89]. Both these techniques are simple, easy to perform also in an outpatient setting, and even non-physician operators could be specifically trained. However, these studies suffer from the absence of the invasive gold standard, and also, the number of patients was relatively low compared to the large number of studies on the IVC available in the literature.

## 11. Future Directions

The aim of our research group is to implement a clinical research program aiming at a thorough characterization of the performance of the edge tracking technique across a range of clinical conditions and healthcare settings [43,90]. Specifically, we are primarily planning the validation of this technique in the acute HF setting to assess the relationship between diuretic response and IVC collapsibility and in the hemodyalisis setting to evaluate the relationship between fluid removal and IVC collapsibility. Finally, our ultimate target is to train our algorithm in a wide and heterogenous cohort of patients affected by heart failure/fluid overload who undergo RHC. We want to perform a prospective multicenter study to build a stable, robust, and reliable algorithm applicable to all clinical settings. Initially, we will evaluate its diagnostic accuracy and then, after an appropriate period of follow up, we will see its prognostic impact dividing the multicenter cohort in two groups: the first followed by repeated IVC edge tracking measurement and secondly the control group. The primary end point will be admission/urgent visit for heart failure relapse: our hope is to be able to reduce as much as possible this adverse event.

## 12. Conclusions

This paper discusses the premises and the scientific evidence that led us to think that a more accurate and reproducible measurement of RAP is necessary in many clinical contexts. A possible solution is represented by our automated “edge-tracking” system of the IVC [30], which is able to analyze a tract of the vessel and estimate accurately the RAP [29] and by other techniques when IVC US assessment is not available [89,90]. Further multicenter studies are planned to evaluate the feasibility and clinical reproducibility of this promising method that could lead to the birth of a new prognostic parameter useful in many clinical settings, especially in the field of heart failure.

## Figures and Tables

**Table 1:** Main studies available with ROC curve evaluation of IVC related indexes (IVC: Inferior Vena Cava; RAP: Right Atrial Pressure).

Author	Number of Patients in the Study	Number of Patients Meeting the Proposed Criteria	Parameter	To Predict	Cut Off	Sensitivity	Specificity
Kircher 1990 [3]	83	47	IVC inspiratory (With “sniff” maneuver)	RAP > 10 mmHg	<50%	87%	82%
Brennan 2007 [4]	102	46	IVC expiratory diameter	RAP > 10 mmHg	>20 mm	73%	85%
Brennan 2007 [4]	102	46	IVC inspiratory (With “sniff” maneuver)	RAP < 10 mm	<12 mm	91%	94%
Moreno 1984 [5]	175	65	IVC Caval Index	RAP < 7 mm	>40%	91%	90%
Vourvouri 2003 [6]	88	20	IVC inspiratory (With “sniff” maneuver)	RAP > 10 mmHg	<50%	87%	100%

## References

[1] Pellicori P., Cleland J.G.F., Zhang J., Kallvikbacka-Bennett A., Urbinati A., Shah P., Kazmi S., Clark A.L. (2016). Cardiac Dysfunction, Congestion and Loop Diuretics: Their Relationship to Prognosis in Heart Failure. Cardiovasc. Drugs Ther..

[2] Rudski L.G., Lai W.W., Afilalo J., Hua L., Handschumacher M.D., Chandrasekaran K., Solomon S.D., Louie E.K., Schiller N.B. (2010). Guidelines for the Echocardiographic Assessment of the Right Heart in Adults: A Report from the American Society of Echocardiography. Endorsed by the European Association of Echocardiography, a registered branch of the European Society of Cardiology. J. Am. Soc. Echocardiogr..

[3] Kircher B.J., Himelman R.B., Schiller N.B. (1990). Noninvasive estimation of right atrial pressure from the inspiratory collapse of the inferior vena cava. Am. J. Cardiol..

[4] Brennan J.M., Blair J.E., Goonewardena S., Ronan A., Shah D., Vasaiwala S., Kirkpatrick J.N., Spencer K.T. (2007). Reappraisal of the Use of Inferior Vena Cava for Estimating Right Atrial Pressure. J. Am. Soc. Echocardiogr..

[5] Moreno F.L., Hagan A.D., Holmen J.R., Pryor T.A., Strickland R.D., Castle C.H. (1984). Evaluation of size and dynamics of the inferior vena cava as an index of right-sided cardiac function. Am. J. Cardiol..

[6] Vourvouri E.C., Schinkel A.F.L., Roelandt J.R.T.C., Boomsma F., Sianos G., Bountioukos M., Sozzi F.B., Rizzello V., Bax J.J., Karvounis H.I. (2003). Screening for left ventricular dysfunction using a hand-carried cardiac ultrasound device. Eur. J. Heart Fail..

[7] Milan A., Magnino C., Veglio F. (2010). Echocardiographic Indexes for the Non-Invasive Evaluation of Pulmonary Hemodynamics. J. Am. Soc. Echocardiogr..

[8] Mesin L., Albani S., Policastro P., Pasquero P., Porta M., Melchiorri C., Leonardi G., Albera C., Scacciatella P., Pellicori P. (2022). Assessment of Phasic Changes of Vascular Size by Automated Edge Tracking-State of the Art and Clinical Perspectives. Front. Cardiovasc. Med..

[9] Long E., Oakley E., Duke T., Babl F.E. (2017). Does Respiratory Variation in Inferior Vena Cava Diameter Predict Fluid Responsiveness: A Systematic Review and Meta-Analysis. Shock.

[10] Orso D., Paoli I., Piani T., Cilenti F.L., Cristiani L., Guglielmo N. (2020). Accuracy of Ultrasonographic Measurements of Inferior Vena Cava to Determine Fluid Responsiveness: A Systematic Review and Meta-Analysis. J. Intensive Care Med..

[11] Si X., Xu H., Liu Z., Wu J., Cao D., Chen J., Chen M., Liu Y., Guan X. (2018). Does Respiratory Variation in Inferior Vena Cava Diameter Predict Fluid Responsiveness in Mechanically Ventilated Patients? A Systematic Review and Meta-analysis. Anesth. Analg..

[12] Blehar D.J., Resop D., Chin B., Dayno M., Gaspari R. (2012). Inferior vena cava displacement during respirophasic ultrasound imaging. Crit. Ultrasound J..

[13] Murphy E.H., Arko F.R., Trimmer C.K., Phangureh V.S., Fogarty T.J., Zarins C.K. (2009). Volume associated dynamic geometry and spatial orientation of the inferior vena cava. J. Vasc. Surg..

[14] Mesin L., Pasquero P., Roatta S. (2020). Multi-directional Assessment of Respiratory and Cardiac Pulsatility of the Inferior Vena Cava From Ultrasound Imaging in Short Axis. Ultrasound Med. Biol..

[15] Mesin L., Roatta S., Pasquero P., Porta M. (2020). Automated Volume Status Assessment Using Inferior Vena Cava Pulsatility. Electronics.

[16] Nakamura K., Tomida M., Ando T., Sen K., Inokuchi R., Kobayashi E., Nakajima S., Sakuma I., Yahagi N. (2013). Cardiac variation of inferior vena cava: New concept in the evaluation of intravascular blood volume. J. Med. Ultrason..

[17] Sonoo T., Nakamura K., Ando T., Sen K., Maeda A., Kobayashi E., Sakuma I., Doi K., Nakajima S., Yahagi N. (2015). Prospective analysis of cardiac collapsibility of inferior vena cava using ultrasonography. J. Crit. Care.

[18] Folino A., Benzo M., Pasquero P., Laguzzi A., Mesin L., Messere A., Porta M., Roatta S. (2017). Vena Cava Responsiveness to Controlled Isovolumetric Respiratory Efforts. J. Ultrasound Med. Off. J. Am. Inst. Ultrasound Med..

[19] Mesin L., Giovinazzo T., D’Alessandro S., Roatta S., Raviolo A., Chiacchiarini F., Porta M., Pasquero P. (2019). Improved Repeatability of the Estimation of Pulsatility of Inferior Vena Cava. Ultrasound Med. Biol..

[20] Sisini F., Toro E., Gambaccini M., Zamboni P. (2015). The Oscillating Component of the Internal Jugular Vein Flow: The Overlooked Element of Cerebral Circulation. Behav. Neurol..

[21] Appleton C.P., Hatle L.K., Popp R.L. (1987). Superior vena cava and hepatic vein Doppler echocardiography in healthy adults. J. Am. Coll. Cardiol..

[22] García-López I., Rodriguez-Villegas E. (2020). Extracting the Jugular Venous Pulse from Anterior Neck Contact Photoplethysmography. Sci. Rep..

[23] Joseph A.A., Voit D., Frahm J. (2020). Inferior vena cava revisited-Real-time flow MRI of respiratory maneuvers. NMR Biomed..

[24] Mesin L., Pasquero P., Roatta S. (2019). Tracking and Monitoring Pulsatility of a Portion of Inferior Vena Cava from Ultrasound Imaging in Long Axis. Ultrasound Med. Biol..

[25] Ermini L., Seddone S., Policastro P., Mesin L., Pasquero P., Roatta S. (2021). The cardiac caval index. Improving non-invasive assessment of cardiac preload. J Ultras Med..

[26] Mesin L., Albani S., Sinagra G. (2019). Non-invasive Estimation of Right Atrial Pressure Using Inferior Vena Cava Echography. Ultrasound Med. Biol..

[27] Wallace D.J., Allison M., Stone M.B. (2010). Inferior Vena Cava Percentage Collapse During Respiration Is Affected by the Sampling Location: An Ultrasound Study in Healthy Volunteers. Acad. Emerg. Med..

[28] Beigel R., Cercek B., Luo H., Siegel R.J. (2013). Noninvasive evaluation of right atrial pressure. J. Am. Soc. Echocardiogr..

[29] Albani S., Pinamonti B., Giovinazzo T., de Scordilli M., Fabris E., Stolfo D., Perkan A., Gregorio C., Barbati G., Geri P. (2020). Accuracy of right atrial pressure estimation using a multi-parameter approach derived from inferior vena cava semi-automated edge-tracking echocardiography: A pilot study in patients with cardiovascular disorders. Int. J. Cardiovasc. Imaging.

[30] Mesin L., Pasquero P., Albani S., Porta M., Roatta S. (2015). Semi-automated tracking and continuous monitoring of inferior vena cava diameter in simulated and experimental ultrasound imaging. Ultrasound Med. Biol..

[31] Magnino C., Omedè P., Avenatti E., Presutti D., Iannaccone A., Chiarlo M., Moretti C., Gaita F., Veglio F., Milan A. (2017). Inaccuracy of Right Atrial Pressure Estimates Through Inferior Vena Cava Indices. Am. J. Cardiol..

[32] Nakamura K., Qian K., Ando T., Inokuchi R., Doi K., Kobayashi E., Sakuma I., Nakajima S., Yahagi N. (2016). Cardiac Variation of Internal Jugular Vein for the Evaluation of Hemodynamics. Ultrasound Med. Biol..

[33] Tokunaga K., Nakamura K., Inokuchi R., Hayase N., Terada R., Tomioka Y., Ikeda T., Kobayashi E., Okazaki H., Sakuma I. (2020). Cardiac Variation of Internal Jugular Vein as a Marker of Volume Change in Hemorrhagic Shock. Shock.

[34] Hung J., Lang R., Flachskampf F., Shernan S.K., McCulloch M.L., Adams D.B., Thomas J., Vannan M., Ryan T. (2007). 3D echocardiography: A review of the current status and future directions. J. Am. Soc. Echocardiogr. Off. Publ. Am. Soc. Echocardiogr..

[35] Huguet R., Fard D., d’Humieres T., Brault-Meslin O., Faivre L., Nahory L., Dubois-Randé J.L., Ternacle J., Oliver L., Lim P. (2018). Three-Dimensional Inferior Vena Cava for Assessing Central Venous Pressure in Patients with Cardiogenic Shock. J. Am. Soc. Echocardiogr..

[36] Pellicori P., Shah P., Cuthbert J., Urbinati A., Zhang J., Kallvikbacka-Bennett A., Clark A.L., Cleland J.G.F. (2019). Prevalence, pattern and clinical relevance of ultrasound indices of congestion in outpatients with heart failure. Eur. J. Heart Fail..

[37] Selvaraj S., Claggett B., Pozzi A., McMurray J.J.V., Jhund P.S., Packer M., Desai A.S., Lewis E.F., Vaduganathan M., Lefkowitz M.P. (2019). Prognostic Implications of Congestion on Physical Examination among Contemporary Patients with Heart Failure and Reduced Ejection Fraction: PARADIGM-HF. Circulation.

[38] Simonavičius J., Sanders van-Wijk S., Rickenbacher P., Maeder M.T., Pfister O., Kaufmann B.A., Pfisterer M., Čelutkienė J., Puronaitė R., Knackstedt C. (2019). Prognostic Significance of Longitudinal Clinical Congestion Pattern in Chronic Heart Failure: Insights From TIME-CHF Trial. Am. J. Med..

[39] Ambrosy A.P., Pang P.S., Khan S., Konstam M.A., Fonarow G.C., Traver B., Maggioni A.P., Cook T., Swedberg K., Burnett J.C. (2013). Clinical course and predictive value of congestion during hospitalization in patients admitted for worsening signs and symptoms of heart failure with reduced ejection fraction: Findings from the EVEREST trial. Eur. Heart J..

[40] Girerd N., Seronde M.F., Coiro S., Chouihed T., Bilbault P., Braun F., Kenizou D., Maillier B., Nazeyrollas P., Roul G. (2018). Integrative Assessment of Congestion in Heart Failure Throughout the Patient Journey. JACC Hear. Fail..

[41] Goonewardena S.N., Gemignani A., Ronan A., Vasaiwala S., Blair J., Brennan J.M., Shah D.P., Spencer K.T. (2008). Comparison of Hand-Carried Ultrasound Assessment of the Inferior Vena Cava and N-Terminal Pro-Brain Natriuretic Peptide for Predicting Readmission After Hospitalization for Acute Decompensated Heart Failure. JACC Cardiovasc. Imaging.

[42] Pellicori P., Carubelli V., Zhang J., Castiello T., Sherwi N., Clark A.L., Cleland J.G.F. (2013). IVC diameter in patients with chronic heart failure: Relationships and prognostic significance. JACC Cardiovasc. Imaging.

[43] Jobs A., Vonthein R., König I.R., Schäfer J., Nauck M., Haag S., Fichera C.F., Stiermaier T., Ledwoch J., Schneider A. (2020). Inferior vena cava ultrasound in acute decompensated heart failure: Design rationale of the CAVA-ADHF-DZHK10 trial. ESC Hear. Fail..

[44] Stewart K.A., Navarro S.M., Kambala S., Tan G., Poondla R., Lederman S., Barbour K., Lavy C. (2020). Trends in Ultrasound Use in Low and Middle Income Countries: A Systematic Review. Int. J. Matern. Child. Heal. AIDS.

[45] Brennan J.M., Ronan A., Goonewardena S., Blair J.E.A., Hammes M., Shah D., Vasaiwala S., Kirkpatrick J.N., Spencer K.T. (2006). Handcarried ultrasound measurement of the inferior vena cava for assessment of intravascular volume status in the outpatient hemodialysis clinic. Clin. J. Am. Soc. Nephrol..

[46] Khandwalla R.M., Birkeland K.T., Zimmer R., Henry T.D., Nazarian R., Sudan M., Mirocha J., Cha J., Kedan I. (2017). Usefulness of Serial Measurements of Inferior Vena Cava Diameter by VscanTM to Identify Patients With Heart Failure at High Risk of Hospitalization. Am. J. Cardiol..

[47] Kimori K., Tamura Y. (2020). Feasibility of Using a Pocket-Sized Ultrasound Device to Measure the Inferior Vena Cava Diameter of Patients With Heart Failure in the Community Setting: A Pilot Study. J. Prim. Care Community Heal..

[48] McHugh M.D., Kutney-Lee A., Cimiotti J.P., Sloane D.M., Aiken L.H. (2011). Nurses’ widespread job dissatisfaction, burnout, and frustration with health benefits signal problems for patient care. Health Aff..

[49] Callender T., Woodward M., Roth G., Farzadfar F., Lemarie J.C., Gicquel S., Atherton J., Rahimzadeh S., Ghaziani M., Shaikh M. (2015). Heart failure care in low- and middle-income countries: A systematic review and meta-analysis. PLoS Med..

[50] Cowie M.R. (2017). The heart failure epidemic: A UK perspective. Echo Res. Pract..

[51] Palardy M., Nohria A., Rivero J., Lakdawala N., Campbell P., Kato M., Griffin L.M., Smith C.M., Couper G.S., Stevenson L.W. (2010). Right ventricular dysfunction during intensive pharmacologic unloading persists after mechanical unloading. J. Card. Fail..

[52] Frea S., Bovolo V., Bergerone S., D’Ascenzo F., Antolini M., Capriolo M., Canavosio F.G., Morello M., Gaita F. (2012). Echocardiographic evaluation of right ventricular stroke work index in advanced heart failure: A new index?. J. Card. Fail..

[53] Frea S., Centofanti P., Pidello S., Giordana F., Bovolo V., Baronetto A., Franco B., Cingolani M.M., Attisani M., Morello M. (2019). Noninvasive Assessment of Hemodynamic Status in HeartWare Left Ventricular Assist Device Patients: Validation of an Echocardiographic Approach. JACC Cardiovasc. Imaging.

[54] Nageh M.F., Kopelen H.A., Zoghbi W.A., Quiñones M.A., Nagueh S.F. (1999). Estimation of mean right atrial pressure using tissue Doppler imaging. Am. J. Cardiol..

[55] Ommen S.R., Nishimura R.A., Hurrell D.G., Klarich K.W. (2000). Assessment of Right Atrial Pressure With 2-Dimensional and Doppler Echocardiography: A Simultaneous Catheterization and Echocardiographic Study. Mayo Clin. Proc..

[56] Toma M., Giovinazzo S., Crimi G., Masoero G., Balbi M., Montecucco F., Canepa M., Porto I., Ameri P. (2021). Multiparametric vs. Inferior Vena Cava–Based Estimation of Right Atrial Pressure. Front. Cardiovasc. Med..

[57] D’Alonzo G.E., Barst R.J., Ayres S.M., Bergofsky E.H., Brundage B.H., Detre K.M., Fishman A.P., Goldring R.M., Groves B.M., Kernis J.T. (1991). Survival in patients with primary pulmonary hypertension. Results from a national prospective registry. Ann. Intern. Med..

[58] Lee W.-T.N., Ling Y., Sheares K.K., Pepke-Zaba J., Peacock A.J., Johnson M.K. (2012). Predicting survival in pulmonary arterial hypertension in the UK. Eur. Respir. J..

[59] Fernandes C.J., Steigner M.L., Piazza G., Goldhaber S.Z. (2019). Collaborative Cardiology and Pulmonary Management of Pulmonary Hypertension. Chest.

[60] Stolfo D., Albani S., Biondi F., De Luca A., Barbati G., Howard L., Lo Giudice F., Tsampasian V., Pasanisi E.M., Airò E. (2020). Global Right Heart Assessment with Speckle-Tracking Imaging Improves the Risk Prediction of a Validated Scoring System in Pulmonary Arterial Hypertension. J. Am. Soc. Echocardiogr. Off. Publ. Am. Soc. Echocardiogr..

[61] Thomas C.A., Anderson R.J., Condon D.F., de Jesus Perez V.A. (2020). Diagnosis and Management of Pulmonary Hypertension in the Modern Era: Insights from the 6th World Symposium. Pulm. Ther..

[62] Murphy S.P., Ibrahim N.E., Januzzi J.L. (2020). Heart Failure with Reduced Ejection Fraction: A Review. JAMAJ. Am. Med. Assoc..

[63] Caughey M.C., Sueta C.A., Stearns S.C., Shah A.M., Rosamond W.D., Chang P.P. (2018). Recurrent Acute Decompensated Heart Failure Admissions for Patients With Reduced Versus Preserved Ejection Fraction (from the Atherosclerosis Risk in Communities Study). Am. J. Cardiol..

[64] Dunlay S.M., Roger V.L., Redfield M.M. (2017). Epidemiology of heart failure with preserved ejection fraction. Nat. Rev. Cardiol..

[65] Ilieșiu A.M., Hodorogea A.S. (2018). Treatment of heart failure with preserved ejection fraction. Advances in Experimental Medicine and Biology.

[66] Ahmed A., Husain A., Love T.E., Gambassi G., Dell’Italia L.J., Francis G.S., Gheorghiade M., Allman R.M., Meleth S., Bourge R.C. (2006). Heart failure, chronic diuretic use, and increase in mortality and hospitalization: An observational study using propensity score methods. Eur. Heart J..

[67] Braunschweig F., Cowie M.R., Auricchio A. (2011). What are the costs of heart failure?. Europace.

[68] Ricciardi E., La Malfa G., Guglielmi G., Cenni E., Micali M., Corsello L.M., Lopena P., Manco L., Pontremoli R., Moscatelli P. (2020). Characteristics of current heart failure patients admitted to internal medicine vs. cardiology hospital units: The VASCO study. Intern. Emerg. Med..

[69] Pellicori P., Kallvikbacka-Bennett A., Khaleva O., Carubelli V., Costanzo P., Castiello T., Wong K., Zhang J., Cleland J.G.F., Clark A.L. (2014). Global longitudinal strain in patients with suspected heart failure and a normal ejection fraction: Does it improve diagnosis and risk stratification?. Int. J. Cardiovasc. Imaging.

[70] Curbelo J., Aguilera M., Rodriguez-Cortes P., Gil-Martinez P., Suarez Fernandez C. (2018). Usefulness of inferior vena cava ultrasonography in outpatients with chronic heart failure. Clin. Cardiol..

[71] Pufulete M., Maishman R., Dabner L., Higgins J.P.T., Rogers C.A., Dayer M., MacLeod J., Purdy S., Hollingworth W., Schou M. (2018). B-type natriuretic peptide-guided therapy for heart failure (HF): A systematic review and meta-analysis of individual participant data (IPD) and aggregate data. Syst. Rev..

[72] Carbone F., Bovio M., Rosa G.M., Ferrando F., Scarrone A., Murialdo G., Quercioli A., Vuilleumier N., Mach F., Viazzi F. (2014). Inferior vena cava parameters predict re-admission in ischaemic heart failure. Eur. J. Clin. Invest..

[73] Veilleux R.P., Wight J.N., Cannon A., Whalen M., Bachman D. (2014). Home diuretic protocol for heart failure: Partnering with home health to improve outcomes and reduce readmissions. Perm. J..

[74] Gundersen G.H., Norekval T.M., Haug H.H., Skjetne K., Kleinau J.O., Graven T., Dalen H. (2016). Adding point of care ultrasound to assess volume status in heart failure patients in a nurse-led outpatient clinic. A randomised study. Heart.

[75] Siddall E.C., Radhakrishnan J. (2012). The pathophysiology of edema formation in the nephrotic syndrome. Kidney Int..

[76] Kapur G., Valentini R.P., Imam A.A., Mattoo T.K. (2009). Treatment of severe edema in children with nephrotic syndrome with diuretics alone—A prospective study. Clin. J. Am. Soc. Nephrol..

[77] Tabel Y., Mungan I., Karakurt C., Kocak G., Gungor S. (2008). Is edema in minimal change disease of childhood really hypovolemic?. Int. Urol. Nephrol..

[78] Dönmez O., Mir S., Özyürek R., Cura A., Kabasakal C. (2001). Inferior vena cava indices determine volume load in minimal lesion nephrotic syndrome. Pediatr. Nephrol..

[79] Gurgoze M.K., Gunduz Z., Poyrazoglu M.H., Dursun I., Uzum K., Dusunsel R. (2011). Role of sodium during formation of edema in children with nephrotic syndrome. Pediatr. Int..

[80] Hypo- and Hypervolemic Edema in Children with Steroid Sensitive Nephrotic Syndrome—PubMed. https://pubmed.ncbi.nlm.nih.gov/25790549/.

[81] Özdemir K., Mir M.S., Dinçel N., Bozabali S., Bulut İ.K., Yilmaz E., Bözeri S. (2015). Bioimpedance for assessing volume status in children with nephrotic syndrome-PubMed. Turk. J. Med. Sci.

[82] Modi P., Glavis-Bloom J., Nasrin S., Guy A., Chowa E.P., Dvor N., Dworkis D.A., Oh M., Silvestri D.M., Strasberg S. (2016). Accuracy of inferior vena cava ultrasound for predicting dehydration in children with acute diarrhea in resource-limited settings. PLoS ONE.

[83] Hwang M., Piskunowicz M., Darge K. (2019). Advanced ultrasound techniques for pediatric imaging. Pediatrics.

[84] Cheriex E.C., Leunissen K.M., Janssen J.H., Mooy J.M., van Hooff J.P. (1989). Echography of the inferior vena cava is a simple and reliable tool for estimation of “dry weight” in haemodialysis patients. Nephrol. Dial. Transplant..

[85] Kaptein M.J., Kaptein J.S., Oo Z., Kaptein E.M. (2018). Relationship of inferior vena cava collapsibility to ultrafiltration volume achieved in critically ill hemodialysis patients. Int. J. Nephrol. Renovasc. Dis..

[86] Agarwal R., Bouldin J.M., Light R.P., Garg A. (2011). Inferior Vena Cava Diameter and Left Atrial Diameter Measure Volume but Not Dry Weight. Clin. J. Am. Soc. Nephrol..

[87] Steinwandel U., Gibson N., Towell A., Rippey J.J.R., Rosman J. (2018). Can a renal nurse assess fluid status using ultrasound on the inferior vena cava? A cross-sectional interrater study. Hemodial. Int..

[88] Pellicori P., Kallvikbacka-Bennett A., Zhang J., Khaleva O., Warden J., Clark A.L., Cleland J.G.F. (2014). Revisiting a classical clinical sign: Jugular venous ultrasound. Int. J. Cardiol..

[89] Pellicori P., Clark A.L., Kallvikbacka-Bennett A., Zhang J., Urbinati A., Monzo L., Dierckx R., Anker S.D., Cleland J.G. (2017). Non-invasive measurement of right atrial pressure by near-infrared spectroscopy: Preliminary experience. A report from the SICA-HF study. Eur. J. Heart Fail..

[90] Chubuchny V., Pugliese N.R., Taddei C., Poggianti E., Spini V., Barison A., Formichi B., Airò E., Bauleo C., Prediletto R. (2021). A novel echocardiographic method for estimation of pulmonary artery wedge pressure and pulmonary vascular resistance. ESC Hear. Fail..

